# Variations in respiratory rate do not reflect changes in tidal volume or minute ventilation after major abdominal surgery

**DOI:** 10.1007/s10877-020-00538-3

**Published:** 2020-06-01

**Authors:** O. F. C. van den Bosch, R. Alvarez-Jimenez, M. M. H. Stam, F. C. den Boer, S. A. Loer

**Affiliations:** 1 Department of Anesthesiology, Amsterdam UMC, VU University, Amsterdam, The Netherlands; 2 Department of Anesthesiology, Zaandam Medical Center, Zaandam, The Netherlands; 3 Department of Surgery, Zaandam Medical Center, Zaandam, The Netherlands

**Keywords:** Respiratory rate, Tidal volume, Minute ventilation, Impedance-based respiratory volume monitor, Abdominal surgery, Postoperative period

## Abstract

Monitoring of postoperative pulmonary function usually includes respiratory rate and oxygen saturation measurements. We hypothesized that changes in postoperative respiratory rate do not correlate with changes in tidal volume or minute ventilation. In addition, we hypothesized that variability of minute ventilation and tidal volume is larger than variability of respiratory rate. Respiratory rate and changes in tidal volume and in minute ventilation were continuously measured in 27 patients during 24 h following elective abdominal surgery, using an impedance-based non-invasive respiratory volume monitor (ExSpiron, Respiratory Motion, Waltham, MA, US). Coefficients of variation were used as a measure for variability of respiratory rate, tidal volume and minute ventilation. Data of 38,149 measurements were analyzed. We found no correlation between respiratory rate and tidal volume or minute ventilation (r^2^ = 0.02 and 0.01). Mean respiratory rate increased within the first 24 h after abdominal surgery from 13.9 ± 2.5 to 16.2 ± 2.4 breaths/min (*p* = 0.008), while tidal volume and minute ventilation remained unchanged (*p* = 0.90 and *p* = 0.18). Of interest, variability of respiratory rate (0.21 ± 0.06) was significantly smaller than variability of tidal volume (0.37 ± 0.12, *p* < 0.001) and minute ventilation (0.41 ± 0.12, *p* < 0.001). Changes in postoperative respiratory rate do not allow conclusions about changes in tidal volume or minute ventilation. We suggest that postoperative alveolar hypoventilation may not be recognized by monitoring respiratory rate alone. Variability of respiratory rate is smaller than variability in tidal volume and minute ventilation, suggesting that adaptations of alveolar ventilation to metabolic needs may be predominately achieved by variations in tidal volume.

## Introduction

Patients are at risk of postoperative respiratory complications after major abdominal surgery [1–5]. Postoperative monitoring of respiratory function on surgical wards usually includes intermittent measurement of respiratory rate and in certain cases also monitoring of peripheral oxygen saturation. Respiratory rate is often included in Early Warning Scores, alerting when respiratory rate is higher or lower than a predefined range [6, 7]. Respiratory rate, however, is influenced by many postoperative factors including pain and medication affecting central regulation of respiration. Opioids can cause bradypnea resulting in hypoventilation, due to change of the CO_2_-response curve [8]. On the other hand, patients may develop atelectasis and an increased respiratory rate may not be accompanied by an increased alveolar ventilation. Indeed, it has previously been shown that low respiratory rate measurements do not reflect episodes of low minute ventilation [9]. To which extent respiratory rate can be used to monitor postoperative respiratory function remains uncertain. As minute ventilation is the product of respiratory rate and tidal volume, we investigated the relation between changes in respiratory rate and changes in tidal volume as well as minute ventilation. We continuously monitored the respiratory rate, changes in tidal volume and in minute ventilation using an impedance-based respiratory volume monitor. This technique has been shown to have an acceptable average error of less than 10% compared to spirometry measurements of tidal volume and minute ventilation in spontaneously breathing patients [10]. This novel monitoring device allows to measure minute ventilation and respiratory rate continuously at the bedside with a high accuracy.

Variability over time in respiratory parameters is a result of complex interactions between cerebral breathing autoregulation and the thoracopulmonary system to allow for optimal gas exchange under specific environmental requirements [11]. As other short-term temporal variations of similar nature (i.e. heart rate variability), breathing variability within normal ranges may be regarded as an indicator of health or disease [12]. For example, reduced respiratory variability has been shown to be associated with illness severity in ICU patients and with asthma severity in children [13, 21]. We therefore were interested to study postoperative variability of respiratory rate, tidal volume and minute ventilation.

The main objectives of this study were to assess the correlations between respiratory rate and changes in tidal volume and minute ventilation in the surgical ward in the first 24 h after abdominal surgery, and to evaluate the variability of these parameters.

## Methods

### Study population

This single center observational study was approved by the Local Research Ethics Committee of the Amsterdam UMC (location VUmc, PUMA study; 2017.304, 7 June 2017). All procedures performed in the study involving human participants were in accordance with the ethical standards of the institutional and/or national research committee and with the 1964 Helsinki declaration and its later amendments or comparable ethical standards. We included 27 consecutive adult patients undergoing elective abdominal surgery with an expected postoperative hospital stay of > 48 h. Patients with a known allergy for adhesives were excluded. An investigator at the pre-operative clinic obtained written informed consent.

### Postoperative care

Patients received either epidural analgesia (bupivacaine 1.25 mg/ml and fentanyl 2.5 µg/ml at a continuous rate of 6–10 ml/h), patient-controlled intravenous analgesia (morphine bolus of 1 mg), or subcutaneous morphine administration at regular intervals based on Numeric Rating Score (NRS) scale. Respiratory rate and pulse oximetry were recorded and patients received supplemental oxygen via nasal cannula to maintain a SaO_2_ of > 95%, or > 91% in severe COPD.

### Data collection

Continuous monitoring of respiratory parameters (respiratory rate [RR], changes in tidal volume [ΔTV] and changes in minute ventilation [ΔMV]) was performed in the postoperative period at the surgical ward. The impedance-based superficial respiratory volume monitor (ExSpiron, Respiratory Motion, Walthan, MA, US) consists of a three-electrode padset placed on the chest of the patient [14]. Measurements were commenced at the post-anesthesia care unit (PACU) before discharge to the general ward. Baseline measurements of TV and MV were taken during a period of normal breathing. The TV and MV were recorded as percentage of the baseline volumes at discharge from the PACU. Continuous data was aggregated to average values per minute. The monitor display was covered to keep hospital personnel blinded to the measurements. However, proper function of the respiratory volume monitor was checked on a daily basis. Data collection was ceased at patients’ request or at discharge from the hospital. After data collection, all data were transferred by an encrypted USB memory stick to a secured desktop computer for further analysis.

### Statistical analysis

Statistical analyses were performed in 38,149 measurements of respiratory rate, changes in tidal volume and minute ventilation using SPSS (Version 22.0, IBM, Armonk, NY) and R (2017, R Core Team, Vienna, Austria). Normally distributed data were presented as mean ± SD and non-parametric data were presented as median with interquartile range. Data were analyzed using a Student’s *t* test or linear regression. Significance was defined as a *p*-value of < 0.05.

To assess low respiratory rate as a predictor for low tidal volume and low minute ventilation, we calculated the sensitivity, specificity, negative predictive value and positive predictive value of respiratory rate, with tidal volume and minute ventilation as outcome measures [9].

Variability over time of respiratory rate, tidal volume and minute ventilation were calculated as a coefficient of variation. This normalizes the variability by dividing the standard deviation by the mean, which allows comparing variability of parameters with different means and units.

## Results

The demographic data of the 27 included patients are summarized in Table 1. We analyzed 38,149 measurements of changes in respiratory rate, tidal volume and minute ventilation.

**Table 1:** Patient demographics

	(*n* = 27)
Median age; years	70 [59–75]
Males	13 (43%)
Body Mass Index; kg m^−2^	26.7 (5.4)
Median ASA classification	2.0 [2.0–3.0]
Pre-operative SpO_2_; %	97 (2.0)
Pre-operative Hb; mmol/L	7.9 (1.2)
ARISCAT	22 (12)
Smoking	9 (33%)
COPD	2 (7.4%)
OSAS	1 (3.7%)
Surgical technique	
Laparoscopy	15 (56%)
Laparotomy	
Lower GI-tract	8 (30%)
Upper GI-tract	1 (3.7%)
Aortic aneurysm repair	3 (11%)
Median duration of surgery; mins	157 [108–205]
Postoperative epidural analgesia	14 (52%)

Data are shown as mean (SD), median [IQR] or frequency (%)

*ASA* American Society of Anesthesiologists score, *ARISCAT* assess respiratory risk in surgical patients in Catalonia score, *COPD* chronic obstructive pulmonary disease, *OSAS* obstructive sleep apnea syndrome, *Hb* hemoglobin level (mmol/L)

**Fig. 1:**
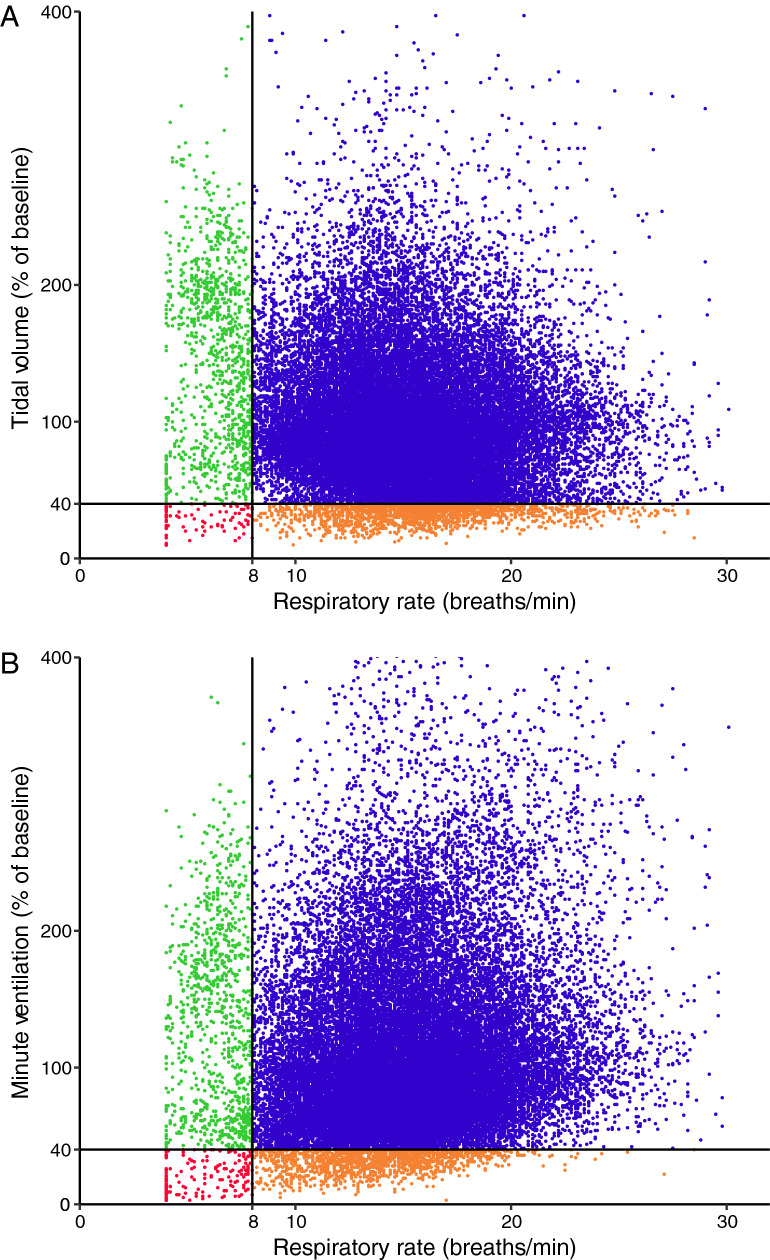
Respiratory rate does not correlate with tidal volume (**a**) and with minute ventilation (**b**) in 24 h after major abdominal surgery. A total of 27 patients with 38,149 paired measurements is shown. There is poor correlation between RR and TV (r^2^ = 0.02) and between RR and MV (r^2^ = 0.01)

We observed no correlation between respiratory rate and tidal volume (r^2^ = 0.02) and between respiratory rate and minute ventilation (r^2^ = 0.01), see Fig. 1.

**Fig. 2:**
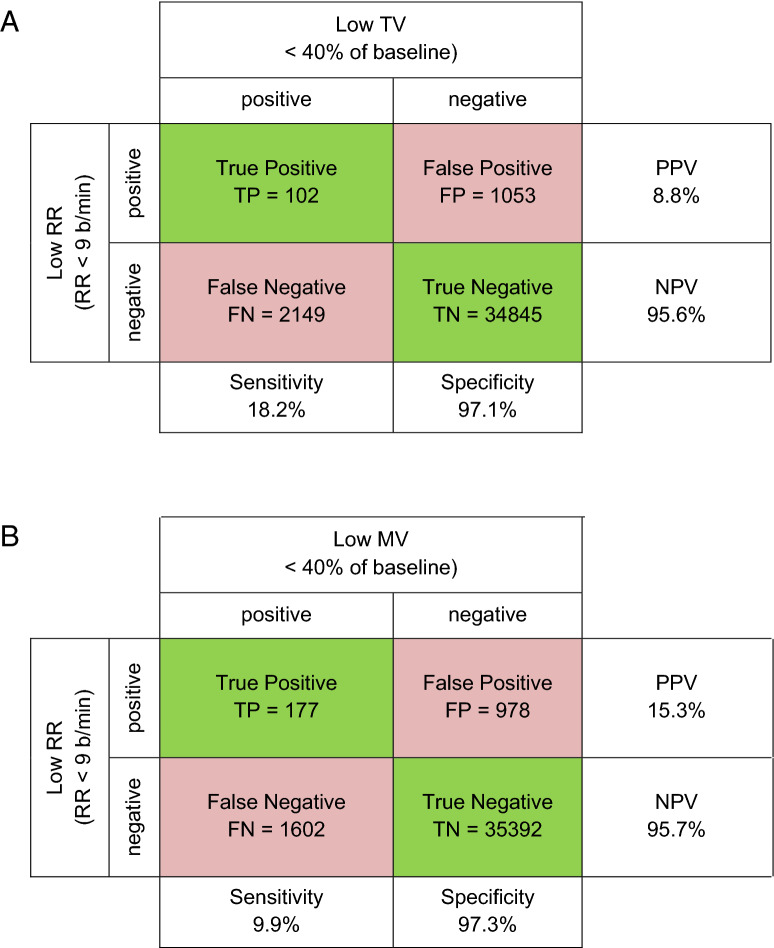
Low respiratory rate (< 9) does not correlate with low tidal volume (< 40% of baseline) (**a**) or low minute ventilation (< 40% of baseline) (**b**) during 24 h after abdominal surgery. A total of 27 patients with 38,149 paired measurements

With respect to the correlation between low respiratory rate (< 9 breath/minute) and low tidal volume measurements (< 40% of baseline), we observed a sensitivity of 4.5%, specificity of 97.1%, negative predictive value of 94.2% and a positive predictive value of 8.8%, see Fig. 2a.

With respect to the correlation between low respiratory rate (< 9 breath/minute) and low minute ventilation measurements (< 40% of baseline), we observed a sensitivity of 9.9% specificity of 9.7%, negative predictive value of 95.7% and a positive predictive value of 15.3%, see Fig. 2b.

**Fig. 3:**
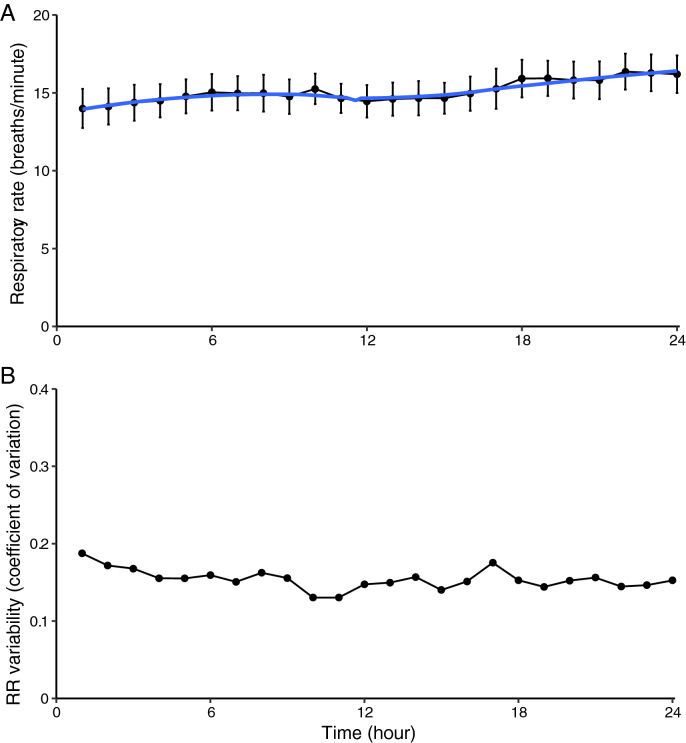
Respiratory rate (RR) during the first 24 h after abdominal surgery. Aggregate data of 27 patients. **a** Average RR per hour. Error bars indicate mean of standard deviation. **b** Variability per hour of RR, depicted as coefficient of variation

**Fig. 4:**
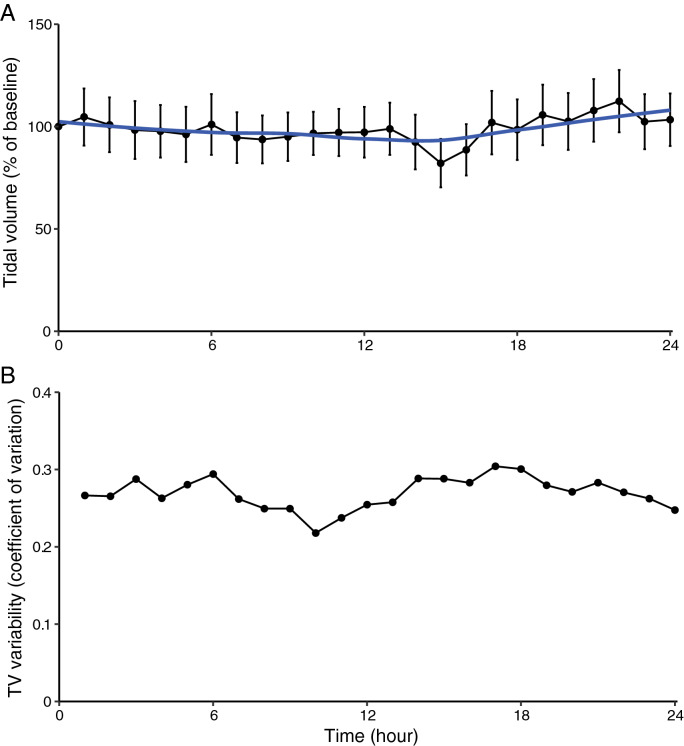
Changes in tidal volume (TV) during the first 24 h after abdominal surgery. Aggregate data of 27 patients. **a** Average TV per hour. Error bars indicate mean of standard deviation. **b** Variability per hour of TV, depicted as coefficient of variation

**Fig. 5:**
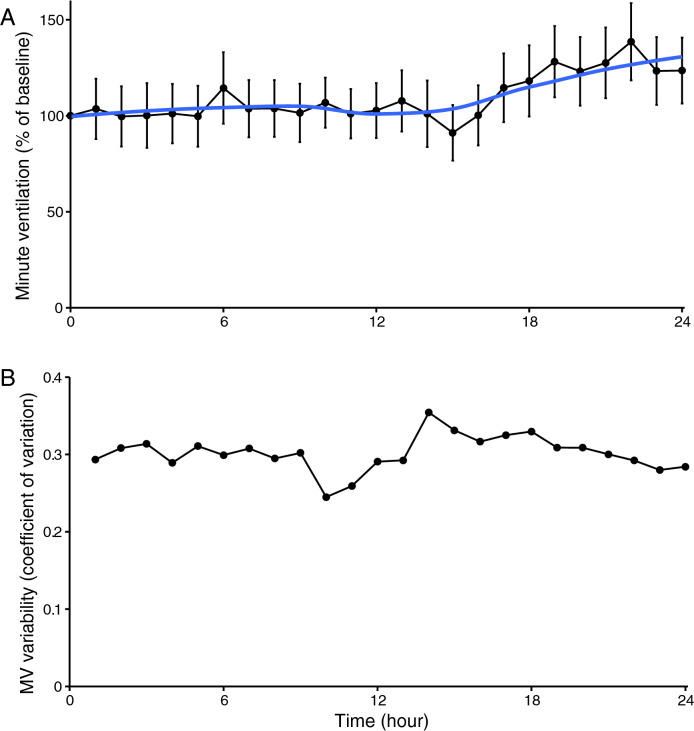
Changes in minute ventilation (MV) during the first 24 h after abdominal surgery. Aggregate data of 27 patients. **a** Average MV per hour. Error bars indicate mean of standard deviation. **b** Variability per hour of MV, depicted as coefficient of variation

The course of respiratory rate, changes in tidal volume and minute ventilation over time, and their variability, are shown in Figs. 3, 4, 5. On average, respiratory rate increases during the 24 h after abdominal surgery. The average respiratory rate in the 1st h at the surgical ward was 13.9 per minute, and increased to 16.1 per minute in the 24th h (p < 0.008). Tidal volume and minute ventilation remained unchanged (*p* = 0.90 and *p* = 0.18, respectively).

**Fig. 6:**
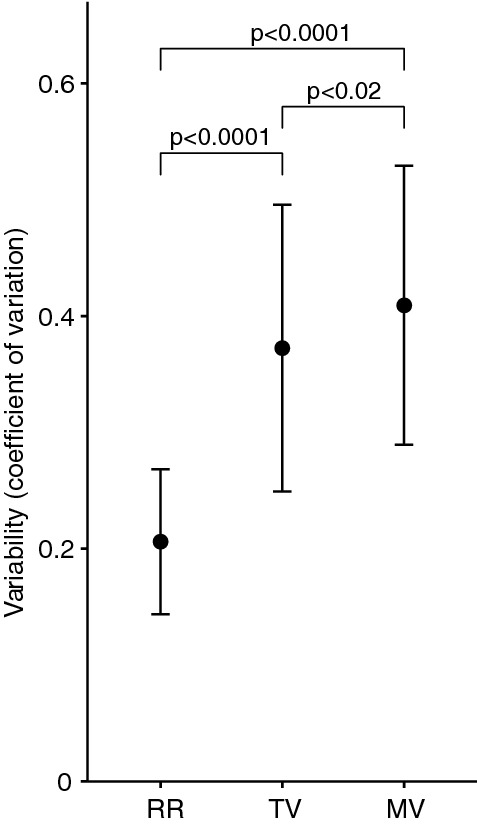
Variability of respiratory parameters (respiratory rate [RR], tidal volume [TV], minute ventilation [MV]) over the first 24 h after elective abdominal surgery in 27 patients. Variability is shown as mean coefficient of variation, error bars indicate standard deviation of variability. Variability of different parameters all differ significantly

There were significant differences between variability of respiratory rate, tidal volume and minute ventilation in the first 24 h (Fig. 6). Over the first 24 h after abdominal surgery, variability of minute ventilation was significantly higher than variability of respiratory rate, mean coefficient of variation 0.41 ± 0.12 vs 0.21 ± 0.06, *p* < 0.0001, and significantly greater than variability of tidal volume 0.41 ± 0.12 vs 0.37 ± 0.12, *p* < 0.02. Variability of tidal volume was also significantly greater than variability of respiratory rate, mean coefficient of variation 0.37 ± 0.12 vs 0.21 ± 0.06, *p* < 0.0001.

## Discussion

Our study showed that measurement of respiratory rate correlates poorly with changes in tidal volume or minute ventilation. At the same time, variability in respiratory rate was smaller than variability in tidal volume and minute ventilation.

### Critical appraisal of methods

We used a non-invasive impedance-based technique to continuously measure respiratory rate and respiratory volume parameters in postoperative patients during 24 h after major abdominal surgery. Earlier studies observed a clinically reasonable accuracy of this respiratory volume monitor in spontaneously breathing patients, with average relative errors for minute ventilation, tidal volume and respiratory of 9.3, 9.0, and 1.8% respectively [9]. This was later confirmed in the pediatric population, where respiratory volume monitor and spirometer measurements were also similar within a 10% error [15]. Also, in intubated patients, measurements correlated well with ventilator settings in cardiac surgery as well as in obese subjects [16, 17]. This makes the monitor well suited to detect early changes in respiration before oxygenation is impaired. A recent review highlighted the promising role of respiratory volume monitoring in management of patients at high risk for respiratory deterioration after surgery [18]. We used a conservative threshold of 40% of baseline to define low tidal volume and minute ventilation, as well as a threshold of < 9 breaths/minute to define low respiratory rate, in line with earlier work [9, 14, 17, 19]. One limitation of our technique regards the measuring technique which only showed relative changes and no absolute values for tidal volume and minute ventilation. Therefore, we only could compare changes in the three target values respiratory rate, tidal volume and minute ventilation. Minute ventilation represents the product of respiratory rate and tidal volume. In our study, minute ventilation was measured and not calculated as a product. Finally, we are not aware of studies measuring 24 h or longer with this method. We, therefore, cannot exclude time-dependent shifts of impedance-based measurement although such shifts appear unlikely.

### Interpretation of results

In accordance with previously published data during procedural sedation in patients undergoing upper gastro-intestinal endoscopic procedures [9], we found no correlation between respiratory rate and tidal volume or minute ventilation. We found a sensitivity of 18.2%, indicating that 81.8% of episodes of low tidal volume (i.e. < 40% of baseline) would have been missed with measurement of respiratory rate alone. Additionally, in periods of hypopnea (< 9 breaths/min), more than 90% would not be accompanied by low tidal volume. This coincides with earlier work demonstrating that respiratory rate is a poor predictor of low minute ventilation [9]. Our results were comparable, with a sensitivity of 9.9% and a specificity of 97.3%. In contrast to previous studies we monitored our patients at least 24 h starting when they left the recovery ward. During our measuring period, mean respiratory rate increased slightly while tidal volume and minute ventilation remained almost unchanged.

Of interest, variability of respiratory rate (0.21 ± 0.06) was significantly smaller than variability of tidal volume (0.37 ± 0.12, *p* < 0.001) and minute ventilation (0.41 ± 0.12, *p* < 0.001). Breathing variability has been shown to be an important marker in a variety of clinical settings. In ICU patients after ceasing sedation, high respiratory rate variability is associated with low organ failure score [13]. Also, during spontaneous breathing trial, greater variability in tidal volume and respiratory rate is associated with increased successful extubation [20]. In children with asthma, lower tidal volume variability at night is associated with a high-risk phenotype (modified Asthma Predictive Index) [21]. In preterm neonates, low tidal volume variability in early life is associated with more rehospitalizations due to respiratory disease [22]. Interestingly, increased breathing variability at rest is also observed in women with elevated blood pressure [23]. Therefore, we conclude that there is a great diversity and individuality in breathing patterns, and within each individual, breathing variability is non-random and controlled by a central neural mechanism or by instability in the chemical feedback loops [11, 24]. Perhaps, adaptation of alveolar ventilation to metabolic needs is predominately achieved by variations in tidal volume rather than respiratory rate. Further research is required to elucidate this mechanism.

In conclusion, monitoring of respiratory rate is insufficient to detect compromise in alveolar ventilation. Changes in respiratory volume could reflect imminent respiratory complications. Thus, to monitor patients at risk, we suggest that respiratory volume monitoring may be preferable above intermittent respiratory rate monitoring, which is the standard of care in many scoring systems.
